# Congenital infection with *Plasmodium malariae*: a rare case of intrauterine transmission in Germany

**DOI:** 10.1186/s12936-025-05331-8

**Published:** 2025-03-20

**Authors:** Sarah Goretzki, Nora Bruns, Anna Daniels, Anne Schönecker, Adela Della Marina, Andrea Gangfuß, Bernd Schweiger, Andreas Schönfeld, Oliver Witzke, Jutta Dedy, Jan Dziobaka, Caroline Holtkamp, Peter-Michael Rath, Ursula Felderhoff-Müser, Christian Dohna-Schwake, Hedda-Luise Verhasselt

**Affiliations:** 1 Department of Pediatrics I, Neonatology, Pediatric Intensive Care, Pediatric Infectiology, Pediatric Neurology, University Hospital Essen, University Duisburg-Essen, Essen, Germany; 2 West German Centre for Infectious Diseases (WZI), University Hospital Essen, University Duisburg-Essen, Essen, Germany; 3 Department of Pediatrics III, Pediatric Cardiology, University Hospital Essen, University Duisburg-Essen, Essen, Germany; 4 Department of Pediatric Neurology, Centre for Neuromuscular Disoders, Centre for Translational Neuro- and Behavioral Sciences, University Duisburg-Essen, Essen, Germany; 5 Department of Pediatric Neurology, Centre for Neuromuscular Disorders, Centre of Translational Neuro- and Behavioral Sciences, University Duisburg-Essen, Essen, Germany; 6 Department of Diagnostic and Interventional Radiology and Neuroradiology, University Hospital Essen, University of Duisburg-Essen, Essen, Germany; 7 Department of Infectious Diseases, West German Centre of Infectious Diseases, University Hospital Essen, University Duisburg-Essen, Essen, Germany; 8 Pharmacy, University Hospital Essen, University of Duisburg Essen, Essen, Germany; 9 Institute of Microbiology, University Hospital Essen, University of Duisburg-Essen, Essen, Germany

**Keywords:** Congenital malaria quartana, *Plasmodium malariae*, Complicated malaria, Artesunate

## Abstract

**Background:**

Malaria remains the leading parasitic disease worldwide with a significant global morbidity and mortality burden. *Plasmodium malariae*, the least prevalent of the five *Plasmodium* species that cause human malaria, has unique characteristics including prolonged prepatent periods and life-long persistance. In non-endemic countries and particular in neonates with coexisting diseases diagnosis and therapy pose challenges.

**Case presentation:**

We report a rare case of severe congenital *P. malariae* malaria in a 2-month-old female infant born in Germany to a Nigerian mother. The infant presented with fever, hepatosplenomegaly, jaundice, and respiratory distress. Initial workup revealed significant haemolysis, hepatopathy, and thrombocytopenia. Microscopic and PCR confirmed *P. malariae*. Shortly after the initial presentation, the infant developed clinical signs of cerebral malaria and organ failure, requiring invasive ventilation, anti-seizure medication, and vasoactive support. Following treatment with intravenous artesunate and oral atovaquone/proguanil, the infant showed significant improvement and was discharged after 36 days (22 days of paediatric intensive care) with a multidisciplinary follow-up plan. At six months post-discharge, she demonstrated stable organ function and mild developmental delay.

**Conclusion:**

The case highlights the diagnostic and therapeutic complexities of life-threatening congenital *P. malariae* infections in non-endemic countries. It underlines the importance of clinicians’ awareness of maternal travel or migration history and individualized treatment strategies. The increasing global mobility necessitates updated guidelines for congenital malaria management even for less likely *P. malariae* infections. Prophylactic measures, early recognition, and multidisciplinary management are critical for improving outcomes for such rare but severe presentations and their long-lasting complications. Possible comprehensive neonatal malaria screening in high-risk populations should be considered in the future.

## Background

Malaria is the most common parasitic disease worldwide, affecting over 80 tropical and subtropical countries across all continents except Australia-Oceania and is the second highest cause of infection-related deaths after tuberculosis [1–3]. The reported number of estimated malaria cases in 2023 is increasing with 263 million (11 million more than 2022) worldwide [2]. The European Centre for Disease Prevention and Control (ECDC) reports 6131 malaria cases in 2022 mainly in France and Germany, 99.8% travel-/migration-related and 86% due to *Plasmodium falciparum* [4].

Globally, five human-pathogenic parasites are known to cause malaria, all transmitted by female *Anopheles* mosquitoes[1]: *Plasmodium falciparum, Plasmodium vivax*, *Plasmodium ovale*, *Plasmodium malariae*, and *Plasmodium knowlesi* [1]. However, recent findings show that *P. ovale* can be genotypically divided into two independent species. The parasites undergo a complex life cycle leading to unique fever patterns [5]: This report will focus on malaria caused by *P. malariae* (Malaria quartana or quartan malaria) with it’s characteristic quartan fever every 72 h [6].

*Plasmodium malariae* is mainly restricted to tropical regions, where it is widespread with higher proportions in Sub-Saharan Africa, South-East Asia and South America [7].

In contrast to other *Plasmodium* species, the prepatent period can last from 16 to 59 days with a relatively low parasitaemia [5]. *Plasmodium malariae* can persist for a lifetime, recurring occasionally especially in episodes of stress and immunosuppression, showing usually a mild or asymptomatic clinical course [6–9]. Only rarely can it lead to a severe, sometimes life-threatening disease, with anaemia, nephritis or pulmonary complications [6, 10, 11]. Congenitally acquired *P. malariae* infections are even less frequently reported, despite an increased risk of infection and parasitaemia during pregnancy. Early signs of transplacental infection are anaemia of the mother or fetus, low birth weight, intrauterine growth restriction and fetal death[12, 13]. Congenital malaria is associated with chronic disease during later life, including diabetes mellitus and heart disease, leaving individuals at higher risk for associated diseases, even after the acute infection [14].

*Plasmodium malariae* is considered to be susceptible to most anti-malarials, including artemisinin-based combinations, and atovaquone-proguanil is the usual treatment when ACT is not available [15–19].

*Plasmodium malariae* is described to be the least likely cause of primary malaria, with very few case presentations and research. Therefore, the treatment of severe cases still presents a true challenge [15].

## Case presentation

### Patient and family history

In the reported case the pregnancy was monitored in Germany and remained unremarkable apart from maternal gestational diabetes and arterial hypertension. There was no evidence of anaemia. All 10 prenatal check-ups, according to the German national guidelines, were unremarkable, as was a routine spontaneous birth in the local hospital. The female infant was born at 37 + 5 weeks’gestation with an APGAR (appearance, pulse, grimace, response, activity, respiration) score of 9/10/10 and birth weight of 3370 g.

Postnatally, trisomy 21 (karyotype: 47, XX + 21) was diagnosed, along with an atrial septal defect (ASD) and a patent ductus arteriosus (PDA). On day five of life, she developed a late-onset sepsis, complicated by respiratory failure and an episode of cor pulmonale without pathogen detection. Treatment included ampicillin and gentamicin, a course of prednisolone, cardiac medication (sildenafil, iloprost, and spironolactone) and invasive ventilation at the local neonatal intensive care unit (NICU). These measures led to clinical improvement and discharge six weeks after birth. There was no evidence of immunodeficiency in the context of trisomy 21, as preliminary and standardized tests were unremarkable.

Family history showed three healthy biological siblings (14-years old sister, 5- and 7-years old brothers), with no evidence of serious illnesses. The children never travelled abroad and there had been no known contact with animals. The 37-year-old Nigerian-born mother last visited her country two years before delivery. She reported a previous infection with *P. falciparum*, which was successfully treated (mother reports chloroquine treatment). The father was also of West African origin and in good health.

### Initial presentation

Two weeks after discharge, the 2-month-old girl and her 5-year-old brother were presented to the paediatrician with fever up to 38.9 °C for 3 days, non-productive cough and clear rhinitis. Primarily a viral respiratory infection was suspected. The symptoms of both children were treated with paracetamol and increased fluid intake. As there was no recovery, the infant was taken to the local children's emergency department two days later.

When presented to the children's emergency department, the infant was in a stable general condition with a body temperature of 38.5 °C. She showed good skin turgor, normal capillary refill time, and no signs of bleeding or meningitis. An icteric skin colour and hepatosplenomegaly as well as moist rales on both sides of her lungs were noticed, with stable vital signs (blood pressure 105/60 mmHg, heart rate 177 bpm, oxygen saturation 99%). The initial laboratory examination revealed the following results: pH 7.21, base excess −6 mmol/L, lactate 4,86 mmol/L, haemoglobin 96 g/L, leucocytes 6,3 × 10⁹/L, thrombocytes 91 × 10⁹/L, C-reactive protein: 19 mg/L, GOT 278 U/L, LDH 607 U/L, bilirubin 161,54 µmol/L and urine status: Bilirubin +  +  + , Protein + . A rapid pathogen diagnosis using PCR for respiratory viruses (Influenza A/B, Respiratory Syncytial Virus (RSV), Severe Acute Respiratory Syndrome Coronavirus 2 (SARS-CoV-2)) and antigen test for streptococci, both from throat swabs, were negative, as well as an extended viral panel on the next day. A sonography of the abdomen in the emergency room showed a hepatosplenomegaly and a mild pericardial effusion of 7 mm.

Because of concerns for impending clinical deterioration due to liver failure, a paediatric infectious disease department at a referral university hospital was consulted including involvement of the. As part of the diagnostic work-up for fever of unknown origin and a Nigerian-born mother, a rapid malaria test (initially without parasite specification) was recommended. The infant was referred to the Children's University Hospital Essen (UME) for further diagnostic workup and potential malaria treatment.

### Further clinical course

During the transport to UME, a first generalized tonic–clonic seizure was observed. On admission to the paediatric intensive care unit (PICU), the infant was breathing spontaneously but was intubated due to clinical deterioration including another generalized seizure despite anti-seizure medication (Midazolam and Levetiracetam). Viral and microbiological testing, as well the chest X-ray revealed no sign of respiratory infection.

The infant was ventilated for 16 days, followed by high-flow-nasal-cannula breathing support for another three days. Pulmonary hypertension subsequently developed again and was successfully treated with Iloprost inhalation. Due to the circulatory failure, the infant was given circulatory support with fluid resuscitation, norepinephrine, and hydrocortisone for six days. The known ASD and PDA were never hemodynamically relevant.

At PICU admission, laboratory findings revealed signs of haemolysis due to malaria (lowest haemoglobin 66 g/L), which required a total of two erythrocyte-transfusions (at admission and after 7 days). Subsequently, all laboratory parameters, including thrombocytopenia (minimum 17 /nl), normalized and signs of haemolysis resolved. Glucose-6-phosphate dehydrogenase deficiency or other haemolytic anaemias were ruled out genetically. There were no nephrological complications, liver enzymes and hepatosplenomegaly normalized after treatment, (maximum GGT 1007 U/L; GOT 243 U/L; GPT 196 U/L – coagulation parameters were normal).

The infant was initially fed via nasogastric tube and later switched to breastfeeding.

**Fig. 1:**
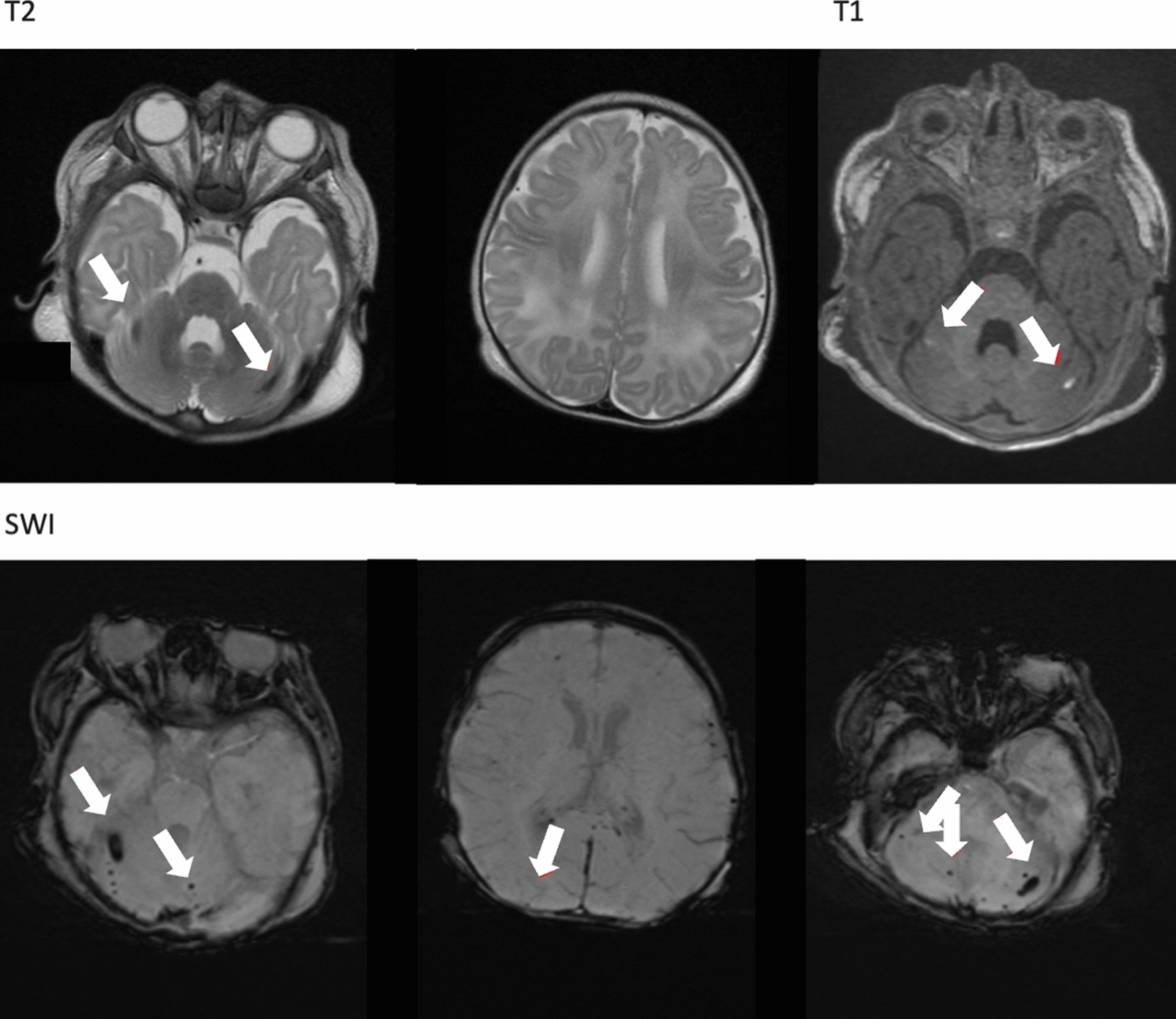
cMRI 2 days after PICU admission T1 hyperintensities in cerebellar hemispheres with corresponding multiple cerebellar hemorrhages in SWI sequences. Bilateral temporo-occipital T2-white matter hyperintensities in subcortical and infratentorial regions, milder in the splenium. Pathologies marked with white arrows

Neurologically, there were signs of cerebral malaria like a prolonged generalized tonic–clonic seizure. As no further seizures occurred, the anticonvulsant therapy with levetiracetam, started at admission, was stopped after 22 days. Cranial Magnetic Resonance Imaging (cMRI) showed multiple small haemorrhages and hypointense lesions in subcortical and infratentorial regions (Fig. 1).

During the hospital stay at UME, extensive neurophysiological, phoniatric, ophthalmological and repeated electroencephalogram (EEG)-recordings revealed no pathologies apart from new-onset mild muscular hypotonia in neurological examinations.

*Plasmodium malariae* was detected in the infant and extensive diagnostics for other infections were negative (Tables 1 and 2). Due to suspected sepsis and rising C-reactive protein (CRP, 4,8 mg/dl), antibiotic treatment with meropenem was started at admission and stopped after 48 h after admission due to negative blood cultures. Antiparasitic therapy consisted of three oral doses of atovaquone/proguanil (320/40 mg/day) and additionally five doses of artesunate intravenously (2.4 mg/kg/dosage) on hour 0, 12, 24, 48 and 72 were started. Since artesunate for treatment of *P. malariae*, is an off-label therapy in Germany, it was conducted in agreement with the German Reference Centre and both parents after informal consent.

**Table 1:** Malaria Diagnostic of mother and infant

Method		Infant lab results	Results for the mother*
Rapid diagnostic test (BinaxNow Malaria, Abbott Laboratories, Chicago, United States)	• Fast (about 30 min) • Orientation test, insufficient as single diagnostic tool [19, 48] • No differences for all malaria species; false positive results for weeks after malaria treatment [19] • false negative RDT results in very high parasitemia due to prozone effect and very low parasitemia (< 200/ul) and very high parasitemia [19]	Positive (T1 line negative (*P. falciparum*), T2 line positive (*P. malariae*, *P. vivax* and/or *P. ovale*)	Negative
Microscopy (Giemsa-stained thin blood smears)	• Primary diagnostic [7, 20–22] • Quantative characteristic of developmental stages of all species [23] (for example reduction after 48 h of therapy) • Repeated testing is necessary to rule out malaria [24, 25]	Positive (parasite count day 1: 0.4%, day 2: 0,35%, day 3: 0,13%, from day 4: 0,00%)	Negative
Molecular screening and typing method using qualitative real-time polymerase chain reaction (PCR) (RealStar Malaria Screen & Type PCR Kit 1.0, Altona Diagnostics GmbH, Hamburg, Germany)	• High sensitivity for detection, but not all are able to differentiate between all species [26, 27] • False negatives with low parasitaemia (for example connatale malaria) and it can lead to false positive (persisting maternal DNA) [27–29]	*P. malariae-*DNA (Ct 18.84, negative day 13 and 71)	Negative on day 3; Positive on day 6 (Ct 32.05)
Serology (indirect immunofluorescence tests)*	• Quantitative and qualitative effectiveness of antimalaria drugs [27−29] • For retrospective issues [48]	Serum: Antibodies against *P. malariae* and *P. fieldi*	Serum: Antibodies against *P. malariae*, *P. falciparum*, and *P. fieldi* Breast milk: Antibodies against *P. malariae* and *P. fieldi* (low titer)

^*^each test was performed at least three times

^**^performed at the Bernhard-Nocht-Institute for Tropical Medicine, the German Reference Centre for Tropical Medicine

**Table 2:** Further diagnostics

Diagnostic	Result
Microbiology	Negative: Tuberculin test and interferon gamma release assay (IGRA) for the detection of *Mycobacterium tuberculosis*, cultures (urine, blood, stool, tracheal secretions), molecular detection of atypical pulmonary pathogens and *Toxoplasma gondii* (tracheal secretions)
Virology	Blood: Negative: Hepatitis A, B, C virus; Cytomegalovirus; Epstein-Barr Virus; Adenovirus; Human Herpesvirus 7/6; Parvovirus B19; Herpes Simplex Virus 1/2; Varicella-Zoster Virus; Enterovirus; human immunodeficiency virus Tracheal secretions: Negative: Influenza A/B and H1N1, rhinovirus, endemic coronavirus, parainfluenza virus, human metapneumovirus, bocavirus, respiratory syncytial virus, enterovirus, adenovirus, human parechovirus
Echocardiography	PDA with left-to-right shunt, ASD, RV hypertrophy, and mild to moderate tricuspid valve insufficiency
Radiology	Normal chest X-ray; cMRI/cCT showed infratentorial and supratentorial microbleeds (see Fig. 1)
Ultrasound	Normal: Skull, abdomen, lungs, and thyroid; initial hepatosplenomegaly regressed over time
Neurophysiology	Normal: AEP, VEP, BERA, and ophthalmological examination
Immunology	Blood smear, differential blood count post-normalization, immunoglobulin levels (including subclasses), Terc Test for severe combined immunodeficiency (SCID), KREC Test (for B-cell defects, e.g., agammaglobulinemia)
Metabolic Diseases	Normal: Organic acids, aminoacids, acylcarnitine-profile and glycosaminoglycans
Others	No evidence of poisoning or medication intoxications

The family was informed in detail about malaria, its symptoms, risks, prophylaxis, and therapy. During the child’s hospital stay, the siblings and father were tested negative for malaria.

Except for haemolysis, the treatment with artesunate was well-tolerated and led to complete elimination of *P. malariae*.

After 36 days (22 days in PICU), the infant was discharged in stable condition. Early intervention therapy and social-medical support was organized, as well as regular checks by the local and university paediatric cardiology, the local neuropaediatric and the University Department of Infectious Disease at UME. Motor and cognitive development, an echocardiography, cMRI and malaria monitoring tests were included.

### Confirmation of the malaria diagnosis

Malaria diagnostics involved multiple tests aiming not only to detect the genus *Plasmodium* spp., but also to determine its species, parasite load, therapy monitoring and potential resistance to common treatments (see Table 1) [7].

**Fig. 2:**
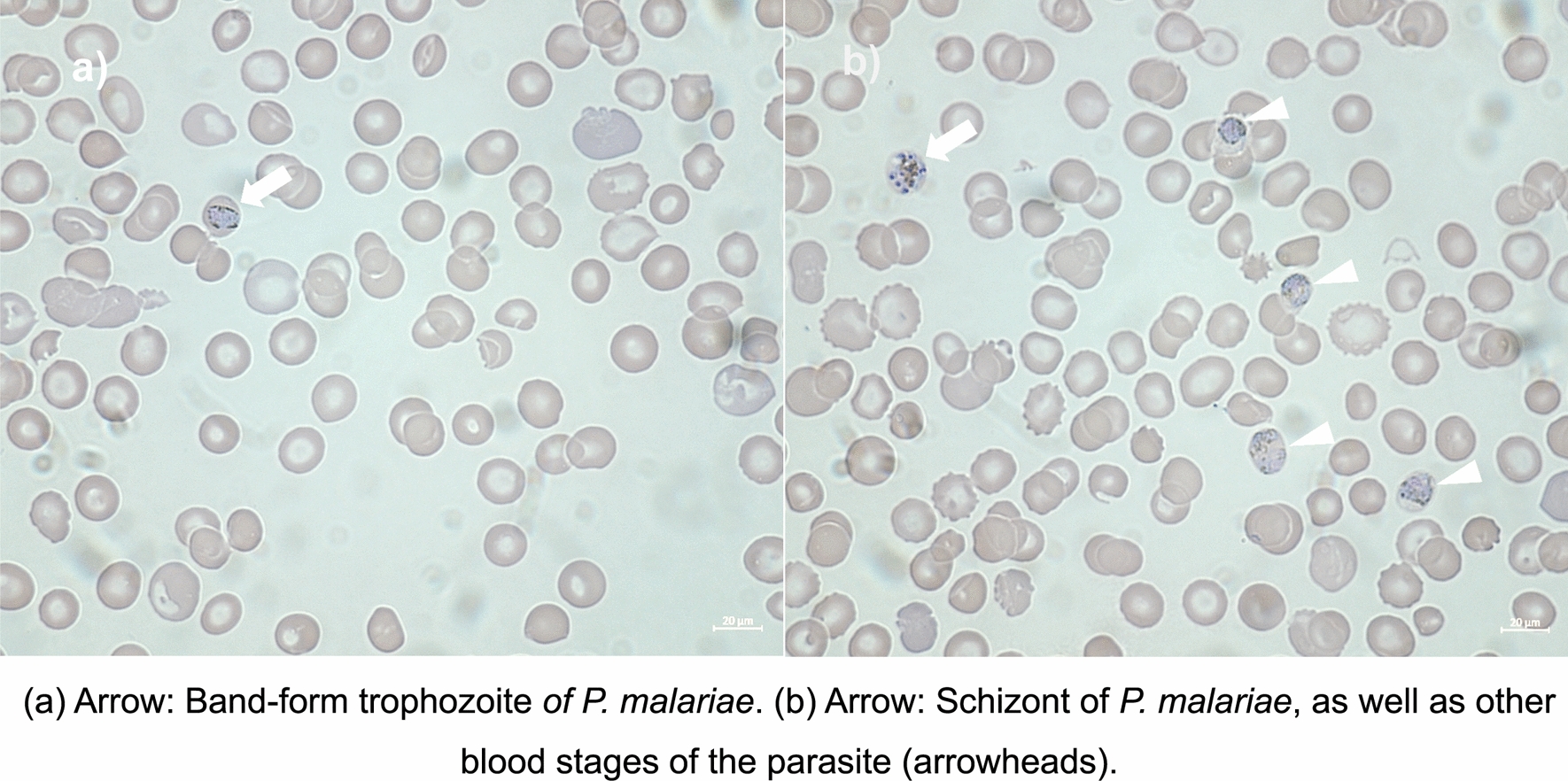
Microscopic of the infant’s blood from two days after PICU admission. **a** Arrow: Band-form trophozoite *of P. malariae*. **b** Arrow: Schizont of *P. malariae*, as well as other blood stages of the parasite (arrowheads)

Microscopy of thin blood smears was repeated on days one, two, three, four, six, 13, 71 and 92 after the initial diagnosis (see Fig. 2). Until day three, parasite load decreased and from day four on *P. malariae* could no longer be detected by microscopy and fluorescence flow cytometry.

Resistance testing is currently not available for routine diagnostics in Germany, and no evidence of P*lasmodium* spp. was detected in the blood of the siblings and their father. Aside from malaria, the diagnostic workup did not identify any additional pathologies, apart from the previously known conditions: trisomy 21, atrial septal defect (ASD), and patent ductus arteriosus (PDA) (see Table 2).

### Follow up

Six months after discharge from PICU the infant showed no relapse for malaria. Bayles neuropsychological development tests depicted a mild motor delay (no sitting without support at 9 month of age), language delay (only 2 words at 6 month of age) and cognitive impairment. The follow-up cMRI showed signs of microangiopathy, as well as small residual lesions post haemorrhages in the cerebellum, most likely due to the malaria. Further follow-up is planned.

## Discussion

Severe congenital *P. malariae* infection as presented, is absolutely rare in a non-endemic country and was transmitted by a Nigerian-born mother after prior infection two years ago. This case highlights the ability of *P. malariae* to persist for extended periods, emphasizing the importance of considering the mother’s travel history to malaria-endemic regions and migration history. Additionally, it is essential to determine whether the patient recalled any previous *P. malariae* infection.

### Malaria in Nigeria

The World Health Organization (WHO) reported 608,000 deaths from malaria worldwide in 2023 with 31% of these occurring in Nigeria [30]. In Nigeria, 90% of malaria-associated deaths affect children under 5 years of age [31]. Congenital malaria is reported with a prevalence of 5.1% to 46.7% in different Nigerian studies—depending on the region and diagnostic method [32–34].

*Plasmodium falciparum (*Malaria tropica) has been the most prevalent, whereas *P. malariae* was found in only 9.8% (26% when using molecular diagnostics) including mostly in mixed malaria infections [35, 36].

### Malaria in pregnancy

In Nigeria, the prevalence of malaria infections during pregnancy has been reported with up to 70%, when sensitive detection methods were used [37]. Malaria infection during pregnancy may lead to impaired placental development and function, causing higher risk of low birth weight, preterm birth, small for gestational age and fetal death [14, 38, 39]. Prevention, early identification, and management of malaria lower those risks, and some endemic countries even screen pregnant women in order to initiate early treatment [26, 34, 38]. As in the described case, pregnant women are rarely asked for malaria symptoms in non-endemic countries, leading to underdetection and putting mothers and children at risk for intrauterine-infections and their complications [40].

### Malaria in early life

The most common cause of quartan malaria also in children is a direct transmission of *P. malariae* through *Anopheles* mosquitoes. Very rarely, it may be transmitted through blood products or non-sterile medical devices [1, 42]. Detection of *Plasmodium* spp. in neonatal blood with no possible postpartum infection defines congenital malaria [43]. There is a direct correlation between maternal parasitaemia, parasitaemia in cord blood or placenta, and congenital infection rate [42]. The congenital infection rate is low, especially in non-endemic countries, and mostly shows unspecific symptoms [40, 44]. In endemic countries congenital malaria is reported with variable incidences for example between 0.1% and 10%, depending on the competence of mother’s immune [41, 45]. There might be a slight increase in congenital malaria globally, due to higher rates of drug resistance, increasing virulence of the *Plasmodium* spp. and possible coinfections with HIV [46]. First symptoms of congenital malaria like fever, hepatosplenomegaly or jaundice mostly occur within 3 to 8 weeks after birth, when maternal antibodies fade [3, 40, 44].

### Challenges in diagnosis of *Plasmodium malariae* in non-endemic regions and neonates

In non-endemic regions, misdiagnosis is even more common in children than in adults and typically more than one physician is contacted before a blood smear is ordered [47, 48]. Furthermore, congenital malaria is very rare in non-endemic countries, with only 81 reported cases in the United States between 1966 and 2005 [49]. At the presented german tertiary neonatal intensive care unit, malaria is not part of the local guidelines for diagnostic work up for sepsis or fever in newborns. However, fever of unknown origin should include malaria testing like a blood smear and PCR [50]. Common symptoms of congenital malaria include fever, anaemia, thrombocytopenia, hepatosplenomegaly, jaundice, regurgitation, diarrhoea, poor feeding or pulmonary distress [43]. All these symptoms may be present in bacterial sepsis, viral infection, or other congenital infections as well [51].

Present experience, including the presented case, highlights the importance to identify newborns at risk and to establish general screening recommendations for congenital malaria. Asking for maternal malaria history (origin, travels, previous illness or anaemia) and testing neonates with unknown symptoms and negative general work up, is essential [34, 43]. Due to the increasing prevalence of immigration from endemic into non-endemic countries, recommendations for screening and treatment should be established also for non-endemic countries.

In the presented case of the mother, both blood films and RDTs failed to detect a *P. malariae* infection and she was treated with chloroquine. However, highly sensitive diagnostic methods, such as PCR, successfully identified the infection. This highlights the importance of considering PCR diagnostics in cases with a history of malaria, migration history, or history of travel to endemic regions, particularly when the mother or child exhibits symptoms.

### Prevention strategies in pregnancy and neonatal care

The latest WHO guideline from November 2024 recommends that pregnant women living in endemic areas receive intermittent preventive treatment with at least three doses, spaced one month apart, of sulfadoxine-pyrimethamine during the second and third trimesters, as it is known to be safe for mother and the unborn child [52–54]. HIV-positive pregnant women are even at a higher risk of severe malaria infections and risk of drug interations, therefore the latest WHO guidelines should be consulted [52–54]. Even for this drug combination resistance has been reported, and new alternatives are needed [43, 55]. Additionally pregnant women should sleep under mosquito-repellent nets [52–54].

### Treatment of congenital infections with *P. malariae*

Literature on therapeutical strategies for congenital malaria is sparce without clear national or international recommendations. It has been reported that newborns with congenital malaria caused by *P. malariae*, regardless of their clinical presentation, can be treated with oral artesunate therapy or quinine [56]. Severe cases of congenital malaria should be treated with intravenous artesunate [57].

### Treatment of infections with *P. malariae*

There are no specific treatment guidelines for *P. malariae* due to its rarity.

However, all forms of severe malaria, including cases in adults, children, congenital infections, and pregnant women, should be treated with intravenous artesunate as the first-line therapy, if available [17, 54]. Following parenteral artesunate, the treatment course may be completed orally.

First choice of oral treatment of *P. malariae* infections are artemisinin-based therapies, including artemether/lumefantrine. Dihydroartemisinin/piperaquine can be used also, for very young children and, to prevent QTc-prolongation, atovaquone/proguanil is used [57–62]. Chloroquine is less suitable as congenital infections are often mixed and there could be resistance to chloroquine reported for *P. falciparum*, *P. vivax* and *P.* malariae [28, 57–62].

### Long-term management

Asymptomatic low-grade *P. malariae* parasitaemia can persist for a long time [6–9]. For that reason, in the treating paediatric infectious disease department, blood smears are analysed after completion of therapy on day 7, 28 as well as 3 and 6 months, even if symptoms are absent.

A possible long-lasting complication after malaria is a neurological impairment [63–68]. Neuropaediatric follow-up should be ensured, especially in complicated malaria. In the presented case, muscular hypotonia was documented for the first time, during severe illness, which could be due to the trisomy 21, the long intensive care unit stay, but also MRI-pathologies including diffuse haemorrhages and white matter changes or a combination of these. In the reported case, cognitive and motor development may be influenced by trisomy 21, and therefore the origins of the residual pathology cannot be differentiated very well. Nevertheless, the microangiopathy observed in the infant’s cMRT can be associated with long-term deficits due to malaria [63–68].

### Immune deficiencies associated with Trisomy 21

Trisomy 21 is associated with congenital immune dysfunctions affecting both the innate and adaptive immune systems [69]. Potential immune deficiencies may contribute to increased severity of malaria infections with impaired parasite control, dysredulation of immune response, compromaised antibody-mediated immunity, T-cell dysfunction and memory response [70]. For this reason, children with risk factors for immundeficiency infections, such as children with trisomy 21, should be evaluated for potential immunological.

As mentioned in Table 2 and the case presentation, in the reported case, evenso the infant had a trisomy 21, there were no laboratory or clinical evidence of immunodeficiency, either initially or during the 6 month of follow-up. The infant demonstrated a normal NK-, B- and T-cell count, no indication of granulocyte dysfunction, a normal differential blood count with regular T-cell subsets and immunoglobulin levels, as well as normal thyroid function and a normal immune response to vaccinations over time (including tetanus, pneumococcal vaccines, and live vaccines).

## Conclusion

The case of a two-month-old with congenital *P. malariae* infection born in Germany, a non-endemic country, is presented with discussion of complexity of diagnosis and management. Despite the increasing prevalence of malaria worldwide, due to migration and global warming, knowledge of rare cases like those from *P. malariae* or congenital malaria in non-endemic countries is limited, leaving them at a higher risk. In the presented case clinical presentation, with signs of severe malaria including neurologic involvement and organ dysfunction underlines the necessity of early recognition of neonates at risk with early diagnosis and treatment.

## Data Availability

No datasets were generated or analysed during the current study.

## Abbreviations

ACT: Artemisinin-based combination therapy
ADV: Adenovirus
AEP: Auditory evoked potentials
APGAR: Appearance, pulse, grimace, activity, respiration
ASD: Atrial septal defect
BERA: Brainstem evoked response audiometry
bpm: Beats per minute
°C: Celsius
CDC: Centers for disease control and prevention
cMRI: Cranial magnetic resonance imaging
CRP: C-reactive protein
Ct: Cycle threshold value
dl: Deciliter
DNA: Desoxyribonucleic acid
ECDC: European Centre for Disease Prevention and Control
EEG: Electroencephalogram
g: Gram
G6PD: Glucose 6 phosphate deficiency
GGT: Gamma-glutamyl transferase
GOT: Glutamate oxaloacetate transaminase
GPT: Glutamate pyruvate transaminase
HIV: Human Immunodeficiency Virus
kg: Kilogram
l: Litre
LDH: Lactate Dehydrogenase
mg: Milligram
mm: Millimetre
mmHg: Millimetres of mercury
mmol: Millimole
NICU: Neonatal intensive care unit
nl: Nanolitre
PCR: Polymerase Chain Reaction
PDA: Patent Ductus Arteriosus
PICU: Paediatric Intensive Care Unit
RDT: Rapid Diagnostic Test
RDW: Red Cell Distribution Width
RSV: Respiratory Syncytial Virus
RV: Right Ventricle
SARS-CoV-2: Severe Acute Respiratory Syndrome Coronavirus 2
SWI: Susceptibility Weighted Imaging
T1 / T2: T1-weighted imaging / T2-weighted imaging
TI: Tricuspid Insufficiency
U: Units
UME: University Medicine Essen
VAI: Viral Antigen Immunoassay
VEP: Visual Evoked Potential
μL: Microlitre

## References

[1] Garcia LS. Malaria. Clin Lab Med. 2010;30:93–129. 20513543 10.1016/j.cll.2009.10.001

[2] World Health Organization. World malaria report 2024: addressing inequity in the global malaria response. Geneva: WHO; 2024.

[3] Schantz-Dunn J, Nour NM. Malaria and pregnancy: a global health perspective. Rev Obstet Gynecol. 2009;2:186–92. 19826576 PMC2760896

[4] European Centre for Disease Prevention and Control. Malaria. In: Annual epidemiological report for 2022. Stockholm: ECDC; 2022.

[5] Centers for Disease Control and Prevention. Parasitemia and lifecycle [Internet]. Available from: https://www.cdc.gov/dpdx/resources/pdf/benchaids/malaria/parasitemia_and_lifecycle.pdf. (Accessed 2024 Jan 3).

[6] Collins WE, Jeffery GM. *Plasmodium malariae*: parasite and disease. Clin Microbiol Rev. 2007;20:579–92. 17934075 10.1128/CMR.00027-07 PMC2176047

[7] Collins WE, Skinner JC, Broderson JR, Pappaioanou M, Filipski V, Sutton BB, et al. The Uganda I/CDC strain of *Plasmodium malariae* in *Aotus lemurinus griseimembra* monkeys. J Parasitol. 1989;75:61–5. 2645394

[8] Grande R, Antinori S, Meroni L, Menegon L, Severini C. A case of *Plasmodium malariae* recurrence: recrudescence or reinfection? Malar J. 2029;18:169. 10.1186/s12936-019-2806-y PMC6515619 31088460

[9] Vinetz JM, Li J, McCutchan TF, Kaslow DC. *Plasmodium malariae* infection in an asymptomatic 74-year-old Greek woman with splenomegaly. N Engl J Med. 1998;338:367–71. 9449730 10.1056/NEJM199802053380605

[10] Langford S, Douglas NM, Lampah DA, Simpson JA, Kenangalem E, Sugiarto P, et al. *Plasmodium malariae* infection associated with a high burden of anemia: a hospital-based surveillance study. PLoS Negl Trop Dis. 2015;9(12): e0004195. 10.1371/journal.pntd.0004195. (**PMID: 26720002**). 26720002 10.1371/journal.pntd.0004195 PMC4697806

[11] Kotepui M, Kotepui KU, Milanez GD. Global prevalence and mortality of severe *Plasmodium malariae* infection: A systematic review and meta-analysis. Malar J. 2020;19:274. 32736635 10.1186/s12936-020-03344-z PMC7395392

[12] Centers for Disease Control and Prevention. Congenital malaria as a result of Plasmodium malariae–North Carolina, 2000. MMWR Morb Mortal Wkly Rep. 2002;51(8):164–5 (**PMID: 11900117**). 11900117

[13] Brabin BJ. An analysis of malaria in pregnancy in Africa. Bull World Health Organ. 1983;61:1005–16. 6370484 PMC2536236

[14] Grunnet LG, Bygbjerg IC, Mutabingwa TK, Lajeunesse-Trempe F, Nielsen J, Schmiegelow C, et al. Influence of placental and peripheral malaria exposure in fetal life on cardiometabolic traits in adult offspring. BMJ Open Diabetes Res Care. 2022;10: e002639. 35379692 10.1136/bmjdrc-2021-002639 PMC8981354

[15] Sutherland CJ. A new window on *Plasmodium malariae* infections. J Infect Dis. 2020;221:864–6. 30855671 10.1093/infdis/jiz103

[16] Betson M, Sousa-Figueiredo JC, Atuhaire A, Arinaitwe M, Adriko M, Mwesigwa G, et al. Detection of persistent *Plasmodium* spp. infections in Ugandan children after artemether-lumefantrine treatment. Parasitology. 2014;141:1880–90. 10.1017/S003118201400033X PMC4255323 24837880

[17] Rutledge GG, Marr I, Huang GKL, Auburn S, Marfurt J, Sanders M, et al. Genomic characterization of recrudescent *Plasmodium malariae* after treatment with artemether/lumefantrine. Emerg Infect Dis. 2017;23:1300–7. 28430103 10.3201/eid2308.161582 PMC5547787

[18] Teo BH, Lansdell P, Smith V, Blaze M, Nolder D, Beshir KB, et al. Delayed onset of symptoms and atovaquone-proguanil chemoprophylaxis breakthrough by *Plasmodium malariae*. PLoS Negl Trop Dis. 2015;9: e0004068. 26485258 10.1371/journal.pntd.0004068 PMC4618945

[19] McMorrow ML, Aidoo M, Kachur SP. Malaria rapid diagnostic tests in elimination settings--can they find the last parasite? Clin Microbiol Infect. 2011;17:1624–31. doi 10.1111/j.1469-0691.2011.03639.x PMC4821879 21910780

[20] Centers for Disease Control and Prevention. Diagnostic procedures for blood specimens [Internet]. 2025. Available from: https://www.cdc.gov/dpdx/diagnosticprocedures/blood/specimenproc.html. (Accessed 2025 Jan 3).

[21] Eberhard ML, Lammie PJ. Laboratory diagnosis of filariasis. Clin Lab Med. 1991;11:977–1010. 1802532

[22] AWMF. Diagnostik-Therapie-Malaria [Internet]. 2021. Available from: https://register.awmf.org/assets/guidelines/042-001l_S1_Diagnostik-Therapie-Malaria_2021-08-verlaengert_01.pdf. (Accessed 2025 Jan 3).

[23] Fuehrer HP, Campino S, Sutherland CJ. The primate malaria parasites *Plasmodium malariae*, *Plasmodium brasilianum* and *Plasmodium ovale* spp.: genomic insights into distribution, dispersal and host transitions. Malar J. 2022;21:138. 10.1186/s12936-022-04151-4 PMC9066925 35505317

[24] Stauffer W, Fischer PR. Diagnosis and treatment of malaria in children. Clin Infect Dis. 2003;37:1340–8. 14583868 10.1086/379074

[25] Başpinar O, Bayraktaroğlu Z, Karsligil T, Bayram A, Coşkun Y. A rare cause of anemia and thrombocytopenia in a newborn: congenital malaria. Turk J Pediatr. 2006;48:63–5. 16562788

[26] Uneke CJ. Diagnosis of *Plasmodium falciparum* malaria in pregnancy in sub-Saharan Africa: the challenges and public health implications. Parasitol Res. 2008;102:333–42. 18038150 10.1007/s00436-007-0782-6

[27] Peters PJ, Thigpen MC, Parise ME, Newman RD. Safety and toxicity of sulfadoxine/pyrimethamine: implications for malaria prevention in pregnancy using intermittent preventive treatment. Drug Saf. 2007;30:481–501. 17536875 10.2165/00002018-200730060-00003

[28] Harrington WE, Duffy PE. Congenital malaria: rare but potentially fatal. Ped Health. 2008;2:235–48.

[29] Grimberg BT. Methodology and application of flow cytometry for investigation of human malaria parasites. J Immunol Methods. 2011;367:1–16. 21296083 10.1016/j.jim.2011.01.015 PMC3071436

[30] World Health Organization. World Malaria Report 2023 [Internet]. 2023. Available from: https://www.who.int/publications/i/item/9789240086173. (Accessed 2025 Jan 3).

[31] Bayode T, Siegmund A. Identifying childhood malaria hotspots and risk factors in a Nigerian city using geostatistical modelling approach. Sci Rep. 2024;14:5445. 38443428 10.1038/s41598-024-55003-x PMC10914794

[32] Obiajunwa PO, Owa JA, Adeodu OO. Prevalence of congenital malaria in Ile-ife. Nigeria J Trop Pediatr. 2005;51:219–22. 15980030 10.1093/tropej/fmi003

[33] Mukhtar MY, Lesi FE, Iroha EU, Egri-Okwaji MT, Mafe AG. Congenital malaria among inborn babies at a tertiary centre in Lagos. Nigeria J Trop Pediatr. 2006;52:19–23. 15927946 10.1093/tropej/fmi044

[34] Falade C, Mokuolu O, Okafor H, Orogade A, Falade A, Adedoyin O, et al. Epidemiology of congenital malaria in Nigeria: a multi-centre study. Trop Med Int Health. 2007;12:1279–87. 17956542 10.1111/j.1365-3156.2007.01931.x

[35] Federal Republic of Nigeria. Nigeria Malaria Indicator Survey 2015 Final Report [Internet]. 2016. Available from: www.DHSprogram.com. (Accessed 2024 Dec 30).

[36] May J, Mockenhaupt FP, Ademowo OG, Falusi AG, Olumese PE, Bienzle U, Meyer CG. High rate of mixed and subpatent malarial infections in southwest Nigeria. Am J Trop Med Hyg. 1999;61:339–43. 10463691 10.4269/ajtmh.1999.61.339

[37] Agomo CO, Oyibo WA, Anorlu RI, Agomo PU. Prevalence of malaria in pregnant women in Lagos. South-West Nigeria Korean J Parasitol. 2009;47:179–83. 19488427 10.3347/kjp.2009.47.2.179 PMC2688802

[38] Satapathy P, Khatib MN, Gaidhane S, Zahiruddin QS, Sharma RK, Rustagi S, et al. Adverse pregnancy outcomes in maternal malarial infection: systematic review and meta-analysis. New Microbes New Infect. 2024;62: 101474. 39286328 10.1016/j.nmni.2024.101474 PMC11403273

[39] Greenwood B. Anti-malarial drugs and the prevention of malaria in the population of malaria endemic areas. Malar J. 2010;9(Suppl 3):S2. 21144082 10.1186/1475-2875-9-S3-S2 PMC3002144

[40] Vottier G, Arsac M, Farnoux C, Mariani-Kurkdjian P, Baud O, Aujard Y. Congenital malaria in neonates: two case reports and review of the literature. Acta Paediatr. 2008;97:505–8. 10.1111/j.1651-2227.2008.00690.x. (**PMID: 18307546**). 18307546 10.1111/j.1651-2227.2008.00690.x

[41] Macgregor JD, Avery JG. Malaria transmission and fetal growth. Br Med J. 1974;3:433–6. 4606777 10.1136/bmj.3.5928.433 PMC1611476

[42] Guertler L. Virus safety of human blood, plasma, and derived products. Thromb Res. 2002;107(Suppl 1):S39-45. 12379292 10.1016/s0049-3848(02)00151-2

[43] Menendez C, Mayor A. Congenital malaria: the least known consequence of malaria in pregnancy. Semin Fetal Neonatal Med. 2007;12:207–13. 17483042 10.1016/j.siny.2007.01.018

[44] Moore KA, Fowkes FJI, Wiladphaingern J, Wai NS, Paw MK, Pimanpanarak M, et al. Mediation of the effect of malaria in pregnancy on stillbirth and neonatal death in an area of low transmission: observational data analysis. BMC Med. 2017;15:98. 28486979 10.1186/s12916-017-0863-z PMC5424335

[45] Romani L, Pane S, Severini C, Menegon M, Foglietta G, Bernardi S, et al. Challenging diagnosis of congenital malaria in non-endemic areas. Malar J. 2018;17:470. 30551740 10.1186/s12936-018-2614-9 PMC6295090

[46] Desai M, ter Kuile FO, Nosten F, McGready R, Asamoa K, Brabin B, Newman RD. Epidemiology and burden of malaria in pregnancy. Lancet Infect Dis. 2007;7:93–104. 17251080 10.1016/S1473-3099(07)70021-X

[47] Goldman-Yassen AE, Mony VK, Arguin PM, Daily JP. Higher rates of misdiagnosis in pediatric patients versus adults hospitalized with imported malaria. Pediatr Emerg Care. 2016;32:227–31. 25322145 10.1097/PEC.0000000000000251 PMC4627851

[48] Kain KC, Harrington MA, Tennyson S, Keystone JS. Imported malaria: prospective analysis of problems in diagnosis and management. Clin Infect Dis. 1998;27:142–9. 9675468 10.1086/514616

[49] Del Castillo M, Szymanski AM, Slovin A, Wong EC, DeBiasi RL. Congenital *Plasmodium falciparum* malaria in Washington. DC Am J Trop Med Hyg. 2017;96:167–9. 28077745 10.4269/ajtmh.15-0630 PMC5239687

[50] Centers for Disease Control and Prevention. Clinical guidance on malaria diagnosis [Internet]. Available from: https://www.cdc.gov/malaria/hcp/clinical-guidance/evaluation-diagnosis.html. (Accessed 2025 Jan 13).

[51] Del Punta V, Gulletta M, Matteelli A, Spinoni V, Regazzoli A, Castelli F. Congenital Plasmodium vivax malaria mimicking neonatal sepsis: a case report. Malar J. 2010;9:63. 20193072 10.1186/1475-2875-9-63 PMC2838910

[52] WHO. A strategic framework for malaria prevention and control during pregnancy in the African Region. Geneva, World Health Organization; 2004. AFR/MAL/04/01.

[53] WHO. Recommendations on the use of sulfadoxine-pyrimethamine (SP) for intermittent preventive treatment during pregnancy (IPT) in areas of moderate to high resistance to SP in the African Region [Internet]. Geneva, World Health Organization; 2005. Available from: http://www.who.int/malaria/publications/atoz/who_sp_statement.pdf. (Accessed 2025 Jan 3).

[54] WHO. Policy brief on malaria in pregnancy [Internet]. Geneva, World Health Organization; 2024. Available from: https://iris.who.int/bitstream/handle/10665/379635/B09146-eng.pdf. (Accessed 2025 Jan 25).

[55] Desai M, Hill J, Fernandes S, Walker P, Pell C, Gutman J, et al. Prevention of malaria in pregnancy. Lancet Infect Dis. 2018;18:e119–32. 29395997 10.1016/S1473-3099(18)30064-1

[56] Orogade AA, Falade CO, Okafor HU, Mokuolu OA, Mamman AI. Clinical and laboratory features of congenital malaria in Nigeria. J Pediatr Infect Dis. 2008;3:181–7.

[57] Avabratha KS, Chettiyar LA, John NP. Oral artesunate for neonatal malaria. J Trop Pediatr. 2010;56:452–3. 20144934 10.1093/tropej/fmq007

[58] Maguire JD, Sumawinata IW, Masbar S, Laksana B, Prodjodipuro P, Susanti I, et al. Chloroquine-resistant *Plasmodium malariae* in South Sumatra. Indonesia Lancet. 2002;360:58–60. 12114045 10.1016/S0140-6736(02)09336-4

[59] Groger M, Veletzky L, Lalremruata A, Cattaneo C, Mischlinger J, Zoleko-Manego R, et al. Prospective clinical trial assessing species-specific efficacy of artemether-lumefantrine for the treatment of *Plasmodium malariae*, *Plasmodium ovale*, and mixed *Plasmodium malaria* in Gabon. Antimicrob Agents Chemother. 2018;62:e01758-e1817. 29311086 10.1128/AAC.01758-17 PMC5826119

[60] Visser BJ, Wieten RW, Kroon D, Nagel IM, Bélard S, van Vugt M, Grobusch MP. Efficacy and safety of artemisinin combination therapy (ACT) for non-falciparum malaria: a systematic review. Malar J. 2014;13:463. 25428624 10.1186/1475-2875-13-463 PMC4258384

[61] Mombo-Ngoma G, Kleine C, Basra A, Würbel H, Diop DA, Capan M, et al. Prospective evaluation of artemether-lumefantrine for the treatment of non-falciparum and mixed-species malaria in Gabon. Malar J. 2012;11:120. 22515681 10.1186/1475-2875-11-120 PMC3393621

[62] Patel AB, Belsare H. Resistant malaria in a neonate. Indian Pediatr. 2002;39(6):585–8. 12084956

[63] Faivre B, Bessis S, Bergounioux J, Duran C, Dinh A, Essid A. A case of postmalaria neurologic syndrome and pediatric literature review. Pediatr Infect Dis J. 2020;39:e325–7. 32932335 10.1097/INF.0000000000002803

[64] Laverse E, Nashef L, Brown S. Neurocognitive sequelae following hippocampal and callosal lesions associated with cerebral malaria in an immune-naive adult. Postgrad Med J. 2013;89:671–2. 23893348 10.1136/postgradmedj-2013-131758

[65] Sousa A, Silva TM, Conceição C, Vieira JP, Gouveia C, Varandas L. Cerebral malaria and cytotoxic lesions of the corpus callosum. Pediatr Infect Dis J. 2023;42:e358–9. 37184269 10.1097/INF.0000000000003963

[66] John CC, Bangirana P, Byarugaba J, Opoka RO, Idro R, Jurek AM, Wu B, Boivin MJ. Cerebral malaria in children is associated with long-term cognitive impairment. Pediatrics. 2008;122:e92–9. 18541616 10.1542/peds.2007-3709 PMC2607241

[67] Zahid A, Mark IT, Gilbertson JR, Johnson DR. Cerebral malaria with extensive subcortical microhemorrhages. Oxf Med Case Reports. 2021;2021:omab028. 10.1093/omcr/omab028 PMC8143660 34055364

[68] Fernando SD, Rodrigo C, Rajapakse S. The “hidden” burden of malaria: cognitive impairment following infection. Malar J. 2010;9:366. 21171998 10.1186/1475-2875-9-366 PMC3018393

[69] Ram G, Chinen J. Infections and immunodeficiency in Down syndrome. Clin Exp Immunol. 2011;164:9–16. 21352207 10.1111/j.1365-2249.2011.04335.x PMC3074212

[70] Su XZ, Xu F, Stadler RV, Teklemichael AA, Wu J. Malaria: Factors affecting disease severity, immune evasion mechanisms, and reversal of immune inhibition to enhance vaccine efficacy. PLoS Pathog. 2025;21: e1012853. 39847577 10.1371/journal.ppat.1012853 PMC11756774

